# Intracardiac Thrombi in Morbus Adamantiades–Behçet in Two Swedish Patients

**DOI:** 10.3390/jcm12165377

**Published:** 2023-08-18

**Authors:** Raffaele Da Mutten, Alexander Borg, Katerina Chatzidionysiou, Ioannis Parodis

**Affiliations:** 1 Division of Rheumatology, Department of Medicine Solna, Karolinska Institutet, 17176 Stockholm, Sweden; raffaele.damutten@uzh.ch (R.D.M.); alexander.borg@ki.se (A.B.); aikaterini.chatzidionysiou@ki.se (K.C.); 2 Department of Gastroenterology, Dermatology and Rheumatology, Karolinska University Hospital, 17176 Stockholm, Sweden; 3 Department of Rheumatology, Faculty of Medicine and Health, Örebro University, 70182 Örebro, Sweden

**Keywords:** Morbus Adamantiades–Behçet, intracardiac thrombus, rheumatology, autoinflammation

## Abstract

Morbus Adamantiades–Behçet (MAB) is an inflammatory disease typically manifesting with oral and genital aphthosis, erythema nodosum, and vasculopathy, and in only around 2%, cardiac involvement. Its prevalence is usually higher along the historic Silk Road, but rarer in Scandinavia where 0.64–4.9 in 100,000 people are affected. We herein present two Swedish patients with cardiac manifestations of Morbus Adamantiades–Behçet. Along with the intracardial thrombi, which both patients presented with, one patient also had cerebrovascular insults leading to visual field deficits as well as involvement of peripheral nerves. Being of Scandinavian origin and showing uncommon symptoms as their initial manifestations of MAB, the 62- and 35-year-old patients presenting herein constitute rare cases.

## 1. Introduction

Morbus Behçet, also known as Morbus Adamantiades–Behçet (MAB), is an inflammatory disease classified as systemic vasculitis [1]. Pathogenetic models that have been proposed include a genetic predisposition that is triggered by infections [1]. Mainly, the Human Leukocyte Antigen (HLA)-B51 allele in the major histocompatibility complex (MHC) is associated with MAB. Also, the prevalence of the disease is considerably more prominent in regions along the historic Silk Road compared with the rest of the world [2,3]. Hence, the occurrence in Scandinavia is rather low with a prevalence ranging from 0.64 to 4.9 in 100,000 people [1,4]. Two relevant sets of criteria have mainly been used. First, the International Study Group criteria were introduced in 1990, showing 91% sensitivity and 96% specificity [5]. In 2014, the International Criteria for Adamantiades–Behçet’s Disease were established [3]. None of these criteria sets include cardiac manifestations as the latter are atypical. Only 2.1% of patients have cardiac symptoms as a first sign [6]. The occurrence of symptoms usually includes oral aphthosis, genital aphthosis, erythema nodosum, and, in 19%, vascular manifestations [3,7,8]. Peripheral nerves may be involved over the course of the disease in only 4.9% of the patients, and the heart in roughly 6% [3,6].

Owing to the rarity of the disease in northern Europe and the even more uncommon presentation with heart involvement at the initial encounter with healthcare, we herein present two Swedish patients diagnosed with MAB who presented with heart involvement among the initial symptoms.

## 2. Case One

**Figure 1:**
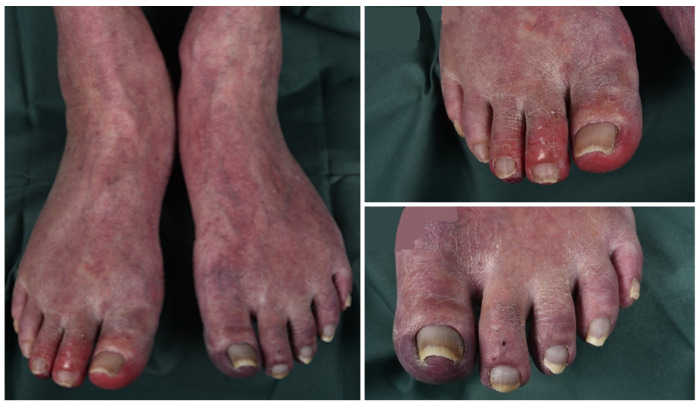
Photographs of case one illustrating signs of vasculopathy.

We present a 62-year-old man from central Sweden who sought care due to general muscular weakness, weight loss, and increased sweating. The patient had been a smoker for fifteen years but had quit a decade ago, and his medical history was unremarkable apart from asthma, benign prostatic hyperplasia, and surgically treated bilateral carpal tunnel syndrome. After the initial workup showing no signs of malignancy and excluding an infection but showing a significantly elevated erythrocyte sedimentation rate (ESR) and C-reactive protein (CRP), he was treated with prednisolone at a daily dose of 40 mg initially which was later tapered to 20 mg daily. Consequently, the inflammatory markers dropped. One month later, he presented again with a cough and dyspnea on slight exertion. Referred to expert care, he presented with white sputum, Raynaud’s phenomenon, muscle weakness, and a loss of vision of the right eye. His legs and feet showed red-purple erythema, indicating a vasculopathy, as illustrated in Figure 1. He also complained of pain in both feet combined with a dorsal extension deficit of the left foot.

The neurological evaluation showed no signs of amyotrophic lateral sclerosis and the examination of the cerebrospinal fluid obtained by lumbar puncture was unremarkable. An electroneurography showed a pronounced sensorimotor axonal polyneuropathy. Electromyographically, myopathic changes were detected at multiple sites and the palsy of the left foot was confirmed. A biopsy of the muscle showed no inflammatory cell infiltrates or vasculitis in the small vessels but hints of neurogenic damage. Computed tomography (CT) and later 18F-fluorodesoxyglucose-Positron Emission Tomography-CT (PET-CT) of the thorax as well as a bronchoscopy showed a CD4^+^/CD8^+^ ratio of 1.5 and no signs of malignancy. Hence, pneumonia and sarcoidosis were deemed unlikely. Further, echocardiography showed an ejection fraction of 11%, general hypokinesia, and a thrombus near the apex measuring 10 × 15 mm.

Ocular diagnostics showed a homonymous right-sided hemianopsia with no signs of intraocular inflammation; it is worth noting that the patient had been under glucocorticoid treatment for several months. The criteria for MAB describe ocular lesions as either anterior uveitis, posterior uveitis, or retinal vasculitis [3]. To this end, it is important to mention that it remains highly unclear what the initial ocular involvement might have been. Possibly, before the treatment with glucocorticoids, there might have been an inflammatory component in the patient’s visual impairment, consistent with the ocular items described in the criteria for MAB. A CT and magnetic resonance imaging (MRI) of the head and neck showed multiple relatively recent left occipital infarctions explaining the loss of vision. Medial and posterior territories showed narrowed lumens, likely due to inflammatory or atherosclerotic factors. To prevent further cerebrovascular insults, dalteparin, which was later substituted with a vitamin K antagonist, and acetylsalicylic acid were initiated.

Antinuclear antibodies (ANA), anti-neutrophil cytoplasmatic antibodies (ANCA), cryoglobulins, antiphospholipid antibodies, autoantibodies related to neurological disease, autoantibodies for myositis diagnostics, complement levels, and creatin kinase were unremarkable. Hence, various common rheumatic diseases including systemic lupus erythematosus were deemed unlikely. Hepatitis and tuberculosis were also ruled out by serology and quantiferon testing.

The clinical picture initially brought to mind a vasculitis, and differential diagnoses included polyarteritis nodosa (PAN). On average, 74% of patients with PAN show peripheral neuropathy and 4–30% show cardiac involvement [9]. After the full diagnostic workup and during the patient’s stay in the inpatient ward, aphthous ulcers in the oral cavity were observed; this was a new symptom for the patient. Later, genetic diagnostics revealed that the patient had the human leukocyte antigen (HLA)-B51 allele. Together with neurological manifestations as well as vascular involvement, resulting in four points in the International Criteria for Behçet’s Disease, the diagnosis MAB with neurological involvement was made [3]. Assuming that ocular involvement might have been a part of the patient’s MAB, accounting for this would yield six points in the criteria set. The patient was subsequently initiated on colchicine and methotrexate, and the anticoagulant therapy was continued.

## 3. Case Two

The second patient was a 35-year-old male, also from central Sweden, who presented with fever, cough, sweating at night, a 3 kg weight loss over the past weeks, and fatigue. Three years before, he had been investigated for erythema nodosum. Yet, no clear etiology was found. One year before, he had repeated episodes of epididymitis, with negative cultures. During the previous winter, the patient had experienced repeated febrile episodes that had been evaluated to be tonsillitides.

At the infectious diseases department, a chest X-ray showed basal infiltrates in the left lung. Inflammatory markers were elevated with a CRP of 110 mg/L and leukocytes of 12 × 10^9^ cells/L. Since urinalysis, virological testing, and four blood cultures did not reveal a pathogen, and antibiotic treatment over the past month had not improved the patient’s condition, infectious etiology was becoming unlikely. As the clinical picture did not resemble sarcoidosis, the working diagnosis was an undifferentiated yet systemic inflammatory disease.

**Figure 2:**
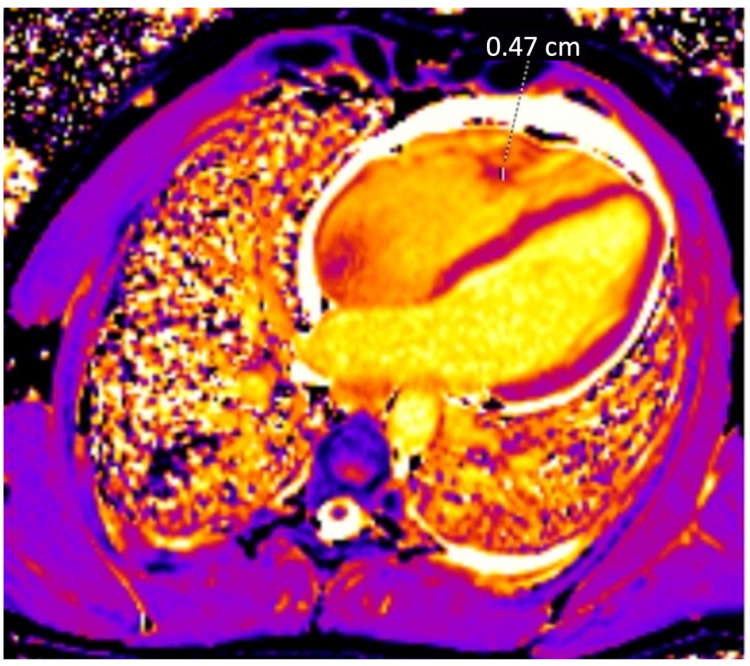
MRI of the heart visualizing a thrombus in the right ventricle.

During investigation, a murmur was heard during heart auscultation, and echocardiography showed vegetations on the mitral and bicuspid valves. Further, an MRI and transesophageal echocardiography were performed. These revealed a pulmonary embolism as well as an intracardiac thrombus of 4.7 mm in diameter (Figure 2). Using computer tomography, splenomegaly and suspected infarction of the right kidney were ascertained.

Upon suspicion of autoimmune endocarditis, oral treatment with prednisolone was initiated, with good results. Moreover, subcutaneous dalteparin was initiated for the thrombi.

Upon further investigation, oral and genital ulcers were described by the patient since childhood, at least six times a year. Genetic testing revealed the presence of HLA-B51. Together with history of pulmonary embolism, renal infarction, intracardial thrombosis, and erythema nodosum, the diagnosis of MAB was ascertained. The high and acute inflammatory activity at presentation and the recurrent epididymitis strengthened the suspicion. Oral and genital ulcers, vascular manifestations, and skin lesions, together give six points in the International Criteria for Behçet’s disease [3].

The glucocorticoids were gradually reduced and discontinued after eight months, while azathioprine and an anti-TNF agent were initiated. Dalteparin was also changed to a vitamin K antagonist, i.e., warfarin. After one year, warfarin was discontinued since a follow-up MRI of the heart showed that the vegetations had resolved. After two years, the anti-TNF agent was also discontinued due to clinical remission.

## 4. Discussion

### 4.1. Clinical Presentation

We herein presented two cases of MAB with uncommon initial presentations. The starting point was, in both cases, an unclear inflammatory condition that was treated with glucocorticoids and later referred to the Rheumatology Department. Yet, it took time to arrive at a clear diagnosis. Partially, this could be due to the low probability of MAB since both patients were not from the Silk Road territory [2]. Also, the presentation with coagulopathy and cardiac involvement is not common. Altogether, this made for a very rare constellation.

Usually, symptoms leading to diagnosis are oral (98%) and genital (74%) aphthosis, as well as erythema nodosum (32%) [3]. Large vein thrombosis, epididymitis, and cardiac manifestations constitute less common complications. However, they still are accounted for more often compared to control patients having at least one major MAB sign of a MAB-mimicking disease. The distribution of cardiac involvement in MAB based on a previously reported series of 52 cases [6] is summarized in Table 1.

The first patient showed oral aphthosis, neurological manifestations, and vascular involvement, resulting in four points in the International Criteria for Behçet’s Disease, or six points if the ocular lesions are accounted for [3]. The second patient presented with oral and genital aphthosis, vascular manifestations, and skin lesions, which sums up to six points [3]. Hence, in both cases, the diagnostic criteria were met. Importantly, extensive investigations were carried out in both cases, during which multiple alternative explanations and mimickers were ruled out, including infectious diseases. Non-bacterial endocarditis, which upon deposition of sterile fibrin and platelets, can result in non-bacterial thrombotic endocarditis, which may be considered a manifestation of MAB, and could have may have been part of the problem in the second case [10,11,12,13].

In a meta-analysis, HLA-B51-positive individuals had a 6-fold higher chance of developing MAB compared with HLA-B51-negative individuals, whereas HLA-B27 positivity also increased the probability of being diagnosed with the disease by almost 2 times [14]. In northern Sweden, the prevalence of HLA-B27-positive individuals is estimated to be 16.6% [15]. Ek et al. [16] stated in a case series of twelve patients in 1993 that only one patient with the HLA-B5 genotype, of which HLA-B51 is a subclass, was not an immigrant [17]. Importantly, the two patients presented herein had no immigration background.

While the Swedish heritage of our patients delayed the diagnostic procedure, these cases emphasize that the disease can also occur in people outside the Silk Road territory, which should not be neglected when patients present with fitting symptoms. Apart from genetic polymorphisms in HLA-B51, interleukin (IL)-10 and IL-10 receptor (IL-10R) have been discussed as factors that may have a role in the pathogenesis of the disease, as have viral and bacterial infections, molecular mimicry, Th1 and Th17 regulation, IL-17, IL-21, IL-23, and endothelial dysfunction [18]. Whether these factors contribute to the disorder that is more or less dependent on heritage or environmental factors has yet to be elucidated. 

### 4.2. Therapy

The 2018 EULAR treatment recommendations suggest both anticoagulant and anti- inflammatory treatment in the case of recurrent deep vein thrombosis, provided that the risk for bleeding is low and pulmonary artery aneurysm is ruled out. However, there is no particular mention of intracardiac thrombosis [19].

Inflammation and coagulopathy are likely linked in MAB [20,21]. An important mechanism seems to be the perivascular neutrophils that facilitate inflammation. Hence, anti-inflammatory treatment is at least equally important as anticoagulation therapy. In fact, a retrospective study of 37 patients has shown that anti-inflammation and anticoagulation treatment combined showed no additional benefit compared with anti-inflammation treatment alone [22]. Furthermore, anticoagulation treatment has, in some cases, been shown to increase the risk of pulmonary artery aneurysm [23].

Eight cases with intracardiac thrombus responded well to glucocorticoids and immunosuppression with either azathioprine or cyclophosphamide, resulting in a resolution of the thrombus and clinical remission in five of those cases [24]. Even though the coagulopathy and cardiac involvement can be treated in many cases, MAB patients with manifestations from the cardiovascular system are characterized by a higher morbidity burden and mortality than those without, and an overall poorer prognosis [6].

In a questionnaire, most American and Israeli rheumatologists as well as about two- thirds of rheumatologists practicing in Turkey, a country among those with the highest prevalence of the disease, stated that they would start immediately with anticoagulation in the case of intracardiac thrombosis [25].

As a matter of fact, with the disease being at the intersections of autoinflammation, autoimmunity, and coagulopathy, therapeutic choices have to be made with caution and desirably by experts.

## 5. Conclusions

We presented two cases of intracardiac thrombosis in patients with Morbus Adamantiades–Behçet, both of them of a Swedish background. A question that arises is whether the incidence of such cases in Nordic countries is currently underreported due to lack of awareness. Surely, in case of an unclear inflammatory condition along with coagulopathy, MAB should be thought of, especially in the absence of other better fitting diagnoses, and prompt relevant genetic investigation. In terms of therapy, a combination of anti-inflammation and anticoagulation may be needed, with the balance between these two compartments of the therapy being, in several cases, delicate.

## Figures and Tables

**Table 1:** Cardiac involvement in MAB, based on a series of 52 patients [6].

Type of Cardiac Involvement	Frequency, No. (%)
Pericarditis	20 (38.5)
Valvular pathology *	14 (26.9)
Intracardiac thrombosis	10 (19.2)
Myocardial infarction	9 (17.3)
Endomyocardial fibrosis	4 (7.7)
Abnormal ECG findings	31 (59.6)

Data based on a series of 52 patients, reported elsewhere [6]. * Including aortic valve insufficiency, mitral valve insufficiency, tricuspid valve insufficiency, mitral valve prolapse, pulmonary valve prolapse, and multiple endocardial involvement. ECG: electrocardiogram; MAB: Morbus Adamantiades–Behçet.

## Data Availability

Not applicable.

## References

[1] Tong B., Liu X., Xiao J., Su G. (2019). Immunopathogenesis of Behcet’s Disease. Front. Immunol..

[2] Davatchi F., Chams-Davatchi C., Shams H., Shahram F., Nadji A., Akhlaghi M., Faezi T., Ghodsi Z., Sadeghi Abdollahi B., Ashofteh F. (2017). Behcet’s Disease: Epidemiology, Clinical Manifestations, and Diagnosis. Expert Rev. Clin. Immunol..

[3] Davatchi F., Assaad-Khalil S., Calamia K.T., Crook J.E., Sadeghi-Abdollahi B., Schirmer M., Tzellos T., Zouboulis C.C., Akhlagi M., International Team for the Revision of the International Criteria for Behçet’s Disease (ITR-ICBD) (2014). The International Criteria for Behçet’s Disease (ICBD): A Collaborative Study of 27 Countries on the Sensitivity and Specificity of the New Criteria. J. Eur. Acad. Dermatol. Venereol..

[4] Mohammad A., Mandl T., Sturfelt G., Segelmark M. (2013). Incidence, Prevalence and Clinical Characteristics of Behcet’s Disease in Southern Sweden. Rheumatology.

[5] Criteria for Diagnosis of Behçet’s Disease (1990). International Study Group for Behçet’s Disease. Lancet.

[6] Geri G., Wechsler B., Thi Huong D.L., Isnard R., Piette J.-C., Amoura Z., Resche-Rigon M., Cacoub P., Saadoun D. (2012). Spectrum of Cardiac Lesions in Behçet Disease: A Series of 52 Patients and Review of the Literature. Medicine.

[7] Ideguchi H., Suda A., Takeno M., Ueda A., Ohno S., Ishigatsubo Y. (2011). Characteristics of Vascular Involvement in Behçet’s Disease in Japan: A Retrospective Cohort Study. Clin. Exp. Rheumatol..

[8] Chen Y., Cai J.-F., Lin C.-H., Guan J.-L. (2019). Demography of Vascular Behcet’s Disease with Different Gender and Age: An Investigation with 166 Chinese Patients. Orphanet J. Rare Dis..

[9] Hernández-Rodríguez J., Alba M.A., Prieto-González S., Cid M.C. (2014). Diagnosis and Classification of Polyarteritis Nodosa. J. Autoimmun..

[10] Mazzoni C., Scheggi V., Mariani T. (2021). Cardiac Involvement in Behçet Disease Presenting as Non-Bacterial Thrombotic Endocarditis: A Case Report. J. Cardiol. Cases.

[11] Kang H.M., Kim G.B., Jang W.-S., Kwon B.S., Bae E.J., Noh C.I., Choi J.Y., Kim Y.J. (2013). An Adolescent with Aortic Regurgitation Caused by Behçet’s Disease Mimicking Endocarditis. Ann. Thorac. Surg..

[12] Lee H.S., Choi W.S., Kim K.H., Kang J.K., Kim N.Y., Park S.H., Park Y., Nam E.J., Yang D.H., Park H.S. (2011). Aseptic Endocarditis in Behçet’s Disease Presenting as Tricuspid Valve Stenosis. Korean Circ. J..

[13] Nassenstein K., Deluigi C.C., Afube T., Schaaf B., Lorenzen J., Bruder O. (2015). Nonbacterial Endocarditis Presenting as a Right Ventricular Tumor in Assumed Behçet’s Disease. Herz.

[14] Khabbazi A., Vahedi L., Ghojazadeh M., Pashazadeh F., Khameneh A. (2019). Association of HLA-B27 and Behcet’s Disease: A Systematic Review and Meta-Analysis. Autoimmun. Highlights.

[15] Bjelle A., Cedergren B., Rantapää Dahlqvist S. (1982). HLA B 27 in the Population of Northern Sweden. Scand. J. Rheumatol..

[16] Ek L., Hedfors E. (1993). Behçet’s Disease: A Review and a Report of 12 Cases from Sweden. Acta Derm. Venereol..

[17] Shenavandeh S., Jahanshahi K.A., Aflaki E., Tavassoli A. (2018). Frequency of HLA-B5, HLA-B51 and HLA-B27 in Patients with Idiopathic Uveitis and Behçet’s Disease: A Case-Control Study. Rheumatology.

[18] Pineton de Chambrun M., Wechsler B., Geri G., Cacoub P., Saadoun D. (2012). New Insights into the Pathogenesis of Behçet’s Disease. Autoimmun. Rev..

[19] Hatemi G., Christensen R., Bang D., Bodaghi B., Celik A.F., Fortune F., Gaudric J., Gul A., Kötter I., Leccese P. (2018). 2018 Update of the EULAR Recommendations for the Management of Behçet’s Syndrome. Ann. Rheum. Dis..

[20] Keser G. (2012). Inflammation-Induced Thrombosis: Mechanisms, Disease Associations and Management. Curr. Pharm. Des..

[21] Emmi G., Silvestri E., Squatrito D., Amedei A., Niccolai E., D’Elios M.M., Della Bella C., Grassi A., Becatti M., Fiorillo C. (2015). Thrombosis in Vasculitis: From Pathogenesis to Treatment. Thromb. J..

[22] Ahn J.K., Lee Y.S., Jeon C.H., Koh E.-M., Cha H.-S. (2008). Treatment of Venous Thrombosis Associated with Behcet’s Disease: Immunosuppressive Therapy Alone versus Immunosuppressive Therapy plus Anticoagulation. Clin. Rheumatol..

[23] Uzun O., Akpolat T., Erkan L. (2005). Pulmonary Vasculitis in Behçet Disease. Chest.

[24] Ben Ghorbel I., Belfeki N., Houman M.H. (2016). Intracardiac Thrombus in Behçet’s Disease. Reumatismo.

[25] Tayer-Shifman O.E., Seyahi E., Nowatzky J., Ben-Chetrit E. (2012). Major Vessel Thrombosis in Behçet’s Disease: The Dilemma of Anticoagulant Therapy—The Approach of Rheumatologists from Different Countries. Clin. Exp. Rheumatol..

